# D1FHS, the Type Strain of the Ammonia-Oxidizing Bacterium *Nitrosococcus wardiae* spec. nov.: Enrichment, Isolation, Phylogenetic, and Growth Physiological Characterization

**DOI:** 10.3389/fmicb.2016.00512

**Published:** 2016-04-14

**Authors:** Lin Wang, Chee Kent Lim, Hongyue Dang, Thomas E. Hanson, Martin G. Klotz

**Affiliations:** ^1^Evolutionary and Genomic Microbiology, Department of Biological Sciences, University of North Carolina at CharlotteCharlotte, NC, USA; ^2^State Key Laboratory of Marine Environmental Science and College of Ocean and Earth Sciences, Xiamen UniversityXiamen, China; ^3^Joint Research Center for Carbon Sink: The Institute of Marine Microbes and Ecospheres, Xiamen University and the Qingdao Institute of BioEnergy and Bioprocess Technology, Chinese Academy of SciencesQingdao, China; ^4^School of Marine Science and Policy, College of Earth, Ocean, and Environment, University of DelawareNewark, DE, USA; ^5^Delaware Biotechnology Institute, University of DelawareNewark, DE, USA; ^6^Evolutionary and Genomic Microbiology, Department of Biology and School of Earth and Environmental Sciences, Queens College, The City University of New YorkFlushing, NY, USA

**Keywords:** ammonia-oxidizing bacteria, enrichment, Jiaozhou Bay, *Nitrosococcus wardiae*

## Abstract

An ammonia-oxidizing bacterium, strain D1FHS, was enriched into pure culture from a sediment sample retrieved in Jiaozhou Bay, a hyper-eutrophic semi-closed water body hosting the metropolitan area of Qingdao, China. Based on initial 16S rRNA gene sequence analysis, strain D1FHS was classified in the genus *Nitrosococcus*, family *Chromatiaceae*, order *Chromatiales*, class *Gammaproteobacteria*; the 16S rRNA gene sequence with highest level of identity to that of D1FHS was obtained from *Nitrosococcus halophilus* Nc4^T^. The average nucleotide identity between the genomes of strain D1FHS and *N. halophilus* strain Nc4 is 89.5%. Known species in the genus *Nitrosococcus* are obligate aerobic chemolithotrophic ammonia-oxidizing bacteria adapted to and restricted to marine environments. The optimum growth (maximum nitrite production) conditions for D1FHS in a minimal salts medium are: 50 mM ammonium and 700 mM NaCl at pH of 7.5 to 8.0 and at 37°C in dark. Because pertinent conditions for other studied *Nitrosococcus* spp. are 100–200 mM ammonium and <700 mM NaCl at pH of 7.5 to 8.0 and at 28–32°C, D1FHS is physiologically distinct from other *Nitrosococcus* spp. in terms of substrate, salt, and thermal tolerance.

## Introduction

**FIGURE 1:**
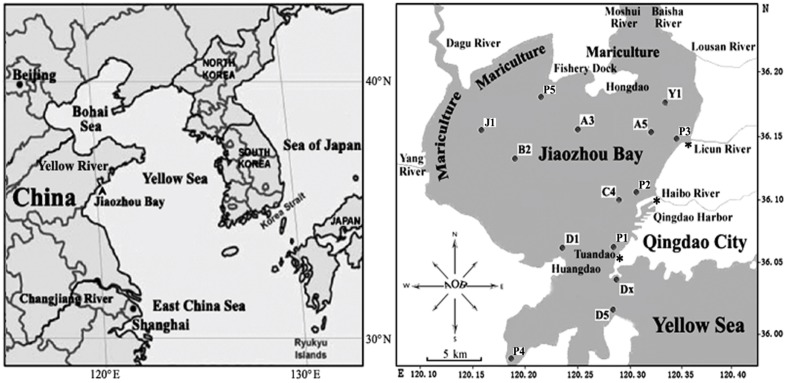
**Map of Jiaozhou Bay and sampling stations**.

Jiaozhou Bay (36°7′24.44′′N 120°14′44.3′′E; **Figure 1**) is a semi-enclosed water body with a surface area of about 390 km^2^ and an average water depth of 7 m, located on the Southwestern coast of Shandong Peninsula, China. It connects to the Chinese Yellow Sea with a narrow mouth, about 2.5 km wide (Shen et al., 2006). The bay is bordered by Qingdao City, a large industrial and agricultural city in North China with a population of approximately 9 million residents. Freshwater is provided to Jiaozhou Bay by numerous tributaries (Yang River, Dagu River, Moshui River, Baishahe River, Wantou River, Loushan River, Banqiaofang River, Licun River, Haipo River, etc.). Most of these rivers have become the canals for industrial, agricultural, and urban wastes and are thus highly enriched in nutrients including concentrated dissolved inorganic nitrogen and inorganic phosphate sources (Shen, 2001). One of the frequently sampled locations is Station D1, (**Figure 1** and Dang et al., 2008, 2009, 2010a). D1 is close to Huangdao industrial park, which includes one of the three largest crude oil storage facilities of China with the capacity to stockpile more than 3 million tons of crude oil as well as a large oil-refining facility with a designed processing capacity of 10 million tons per year (Shen et al., 2006; Dang et al., 2009, 2010a). Huangdao industrial park also contains one of the largest sea terminals for the shipment of crude and refined oil. Despite the advanced eutrophic state of the water column, the much less impacted sediments revealed a highly diverse community of ammonia-oxidizing bacteria (AOB) that facilitate aerobic (Dang et al., 2010b) and anaerobic (Dang et al., 2010a) removal of ammonium. This was the impetus for us to use sediments from this unique environment to search for unknown representatives of marine AOB.

Obligate aerobic chemolithotrophic AOB utilize ammonium as the sole source for energy and reducing power for growth by oxidizing its non-protonated form, ammonia, to nitrite with hydroxylamine as the intermediate. While initially defined primarily by differences in cell morphology and physiological characteristics (Watson and Mandel, 1971), the advent of 16S rRNA gene phylogeny led to the definition of three genera of AOB (Head et al., 1993), which was largely confirmed by the sequencing of available representatives in pure culture (Arp et al., 2007; Campbell et al., 2011). While the betaproteobacterial AOB (beta-AOB) in the family *Nitrosomonadaceae* represent two genera with numerous species (Urakawa et al., 2015), *Nitrosococcus* is the only genus of gammaproteobacterial AOB (gamma-AOB; Campbell et al., 2011). In the past, research of the ecology and phylogeny of AOB has almost entirely focused on the beta-AOB because this group is abundant in all oxic environments, accessible to molecular ecological analysis because the evolutionary histories of enzymes that are key to ammonia catabolism are monophyletic and congruent with 16S rRNA gene-based phylogeny, and beta-AOB usually grow faster than gamma-AOB (Kowalchuk and Stephen, 2001; Campbell et al., 2011). Gamma-AOB in the genus *Nitrosococcus* are adapted to and restricted to marine environments (**Table 1**; Ward and O’Mullan, 2002) and strains representing three species are being maintained in pure culture (Campbell et al., 2011).

**Table 1:** Genome-sequenced gammaproteobacterial AOB species.

	Species/Strain	Date of Isolation	Isolated by	Origin of site
Gamma-AOB	*Nitrosococcus oceani*		
	C-107	1957	S. W. Watson	North Atlantic
	AFC 132	1966	A. F. Carlucci	South Pacific (13°S,78°W)
	AFC27	1964	A. F. Carlucci	North Pacific (43°N,155°W)
	C-27	1964	S. W. Watson	Barbados Harbor
	*Nitrosococcus halophilus*		
	Nc4	1990	Harms/Koops	Salt lagoon, off Sardinia
	*Nitrosococcus watsonii*		
	C-113	1966	S. W. Watson	Red Sea

As recently summarized (Campbell et al., 2011), the type strain of the genus *Nitrosococcus* was originally described by Winogradsky (1892), renamed ‘*Micrococcus nitrosus*’ by Migula (1900) and finally established as *N. nitrosus* (Buchanan, 1925). The binomial was approved as valid by Skerman et al. (1980); however, by then the culture was already lost thereby rendering the genus without a validly described type strain in culture (Campbell et al., 2011). Because Winogradsky enriched *N. nitrosus* reportedly from freshwater, marine strain D1FHS isolated into pure culture (from station **D1** in Jiaozhou Bay, **F**ermented in a bioreactor and cultured in the presence of **H**ydroxylamine at 700 mM NaCl instead of the typical 500 mM in mineral **S**alts media) cannot be assigned to the valid type species of the *Nitrosococcus* genus. The characterization of its properties indicates that D1FHS is physiologically and taxonomically distinct from other *Nitrosococcus* spp. leading us to propose it as the type strain of *N. wardiae* spec. nov. Strain D1FHS has been submitted to the Japan Collection of Microorganisms (JCM) and the American Type Culture Collection (ATCC) for deposit.

## Materials and Methods

### Sample Collection, Cultivation, and Screening of the Enrichment

Sediment samples were collected from station D1 (120.16438°E, 36.00879°N) and from 13 other stations of Jiaozhou Bay (**Figure 1**) on October 26, 2008, as described previously by Dang et al. (2009). Briefly, surface sediment subcore samples were collected from 14 stations including D1 (**Figure 1**) in summer 2008 using a stainless steel 0.05-m^2^ Gray O’Hara box corer. Replicate samples were taken from inside the box core reaching to a depth of 5 cm, placed in sterile Ziploc bags and stored on dry ice and at 80°C after returning to the laboratory. Water was collected at each of the 14 sampling sites and temperatures, dissolved oxygen, salinity, and pH of all surface sediments were measured (**Table 2**). Only sediment samples from station D1 were used for this study.

**Table 2:** Environmental factors for Jiaozhou Bay sampling on 2008-10-26.

	D1	J1	P5	A5	P3
Time	10:45	12:00	12:30	13:40	14:35
Longitude (°E)	120.13418	120.09000	120.11576	120.18332	120.20463
Latitude (°N)	36.04186	36.09500	36.11724	36.08859	36.09851
Water depth (m)	8.0	2.0	3.2	6.3	5.1
Surface seawater temperature (°C)	18.5	16.2	18.5	18	20
Surface seawater salinity (%)	3.5	3.0	3.2	3.3	3.3
Sediment temperature (°C)	14.8	16	16.8	16.8	17
pH (surface seawater)	8.13	8.09	8.13	8.0	7.95
DO (surface seawater; mg/L)	11.31	10.85	10.96	10.29	12.33

Enrichments were started by mixing 0.5 g of frozen sediment into 100 mL of ammonia mineral salts media [AMS: 700 mM NaCl, 12.5 mM (NH_4_)_2_SO_4_, 30 mM MgSO_4_, 20 mM MgCl_2_, 10 mM CaCl_2_, 10 mM KCl, 0.2 mM NaCO_3_, 3.0 mM NaHCO_3_, 0.09 mM K_2_HPO_4_, 3 μM chelated iron, 0.4 μM NaMoO_4,_ 1.0 μM MnCl_2_, 0.008 μM CoCl_2_, 0.35 μM ZnSO_4_, 0.08 μM CuSO_4_, phenol red 0.5% w/v] amended with hydroxylamine at 100 μM final concentration (AMS-H) in a 250 ml Erlenmeyer flask (Pyrex, USA) and maintained as standing batch cultures at 30°C, in the dark, without shaking (Campbell et al., 2011). Phenol-red was used as a pH indicator and pH was adjusted daily to 7.5∼8.0 using 0.25 M K_2_CO_3_. Batch cultures were propagated monthly by transferring 5 mL of culture into 100 mL of fresh AMS-H medium using a sterile pipette (Corning, USA). Hydroxylamine was added weekly to a final concentration of 200 μM to eliminate susceptible bacteria and archaea (Campbell et al., 2011). Nitrite (NO_2_^-^) was measured in spent medium samples (1.3 mL) as an indicator of catabolic activity of nitrifying bacteria by the sequential addition of 0.5 mL of 1% sulfanilamide and 0.5 mL of 0.02% N-(l-napthyl) ethylenediamine dihydrochloride using sterile medium as a control (Nicholas and Nason, 1957; Koops et al., 1990). Following incubation for 20 min at room temperature, absorbance at 543 nm was measured using a Nanodrop 200C spectrophotometer (Thermo scientific Inc.). Contamination with heterotrophs potentially introduced during handling and transfer of cultures was assessed by plating an aliquot on LB agar and incubation at 35°C.

Biomass was harvested from 200 mL of D1 enrichment in exponential phase (based on NO_2_^-^ measurement) by centrifugation at 4,300 × g for 15 min using a Sorvall Evolution centrifuge and SS-34 rotor (Thermo Fisher Scientific, USA). The resulting cell pellet was resuspended in water to a volume of 200 μl for metagenomic DNA extraction using the MP FastDNA Spin Kit (MP Biomedicals, USA) and a MP FastPrep^®^-24 Instrument (MP Biomedicals, USA; Poret-Peterson et al., 2008). Genomic DNA, primers targeting universal and gammaproteobacteria-16S rRNA-specific sequences of the 16S rRNA gene (Campbell et al., 2011) as well as primers specific for detection of the *hao*A gene from gamma-AOB (Schmid et al., 2008) and the polymerase chain reaction (PCR) were used to generate inserts for clone libraries (**Table 3**). Amplicons (universal 16S ∼1470 bp, gamma-16S ∼1500 bp, and gamma-*hao*A ∼1220 bp) were generated using GoTaq Green Master^®^ Mix (Promega, USA) on a gradient mastercycler (Eppendorf, Germany) following an optimized PCR protocol (30 cycles of 1 min at 95°C, 1 min at annealing temperature (*T*_m_): 55°C for universal 16S, 56°C for gamma 16S and 60°C for gamma *hao*A, 1 min extension time at 72°C). For each 50-μl PCR reaction, 2 μl of extracted D1 metagenomic DNA was used as the template. The PCR using gamma-16S rRNA-gene primers generated products of the expected length; therefore, PCR products obtained with universal 16S rRNA and gamma-*hao*A gene primers were gel-purified (1.5% low-melt agarose) and ligated into pCR^®^2.1-TOPO TA vectors (Invitrogen, USA). The overnight ligation products were used to transform TOP10 competent cells for clone library construction. Forty randomly picked recombinants from each clone library were selected using X-Gal-LB plates with 25 μg/ml kanamycin (Dang et al., 2008, 2009, 2011; Campbell et al., 2011). Plasmids were extracted using the alkaline mini-prep method (Wizard Plus SV Minipreps DNA Purification Systems; Promega, USA) and inserts were sequenced with vector primers, M13F and M13R (**Table 3**; DNA Core Facility, University of Louisville). Insert sequences were trimmed, aligned using MUSCLE (Edgar, 2004) provided at the EMBL-EBI webserver^1^ and analyzed for closest-matches with sequences deposited in GenBank using the BLASTN program (Altschul et al., 1997).

**Table 3:** Primers used in this study.

Name	Sequence (5′–3′)	Used for	Reference
M13F	GTAAAACGACGGCCAG	Sequencing	–
M13R	CAGGAAACAGCTATGA	Sequencing	–
27F	AGAGTTTGATCMTGGCTCAG	PCR	Weisburg et al., 1991
1492R	GGTTACCTTGTTACGACTT	PCR	Turner et al., 1999
Gamma-haoA fwd	YTGYCAYAAYGGRGYNGAYCAYAAYGAGT	PCR	Campbell et al., 2011
Gamma-haoA rev	TTRTARWGCTKGAKSANRTGMTGYTCCCACAT	PCR	Campbell et al., 2011
Gamma-16S fwd (F116s)	CAATCTGAGCCATGATCAAAC	PCR	This study
Gamma-16S rev (R16s2)	CCTACGGCTACCTTGTTACG	PCR	This study

### Isolation of D1FHS into Pure Culture, DNA Extraction, Genome Sequencing, and Annotation

Sample aliquots of 0.1 ml were sequentially diluted, plated on sterile semi-solid (0.3% agar) AMS-H medium and incubated in the dark at 30°C (Tittsler and Sandholzer, 1936). After 2 months, visible single colonies were picked and transferred into a 6-well plate with 5 ml liquid AMS-H media. Subsamples (2.5 ml) of wells that turned yellow, indicating acidification due to nitrous acid production, were transferred into 250 ml Erlenmeyer flasks with 100 ml AMS-H media. Enriched culture (20 mL) was used to inoculate a benchtop bioreactor (BioFlo^®^/CelliGen^®^ 115, New Brunswick/Eppendorf, Germany) with 1 L working volume of AMS-H media sterilized by autoclaving (121°C, 20 min, 100 Kpa, 15 psi). The chemostat, operated in batch culture mode, was incubated in the dark at 28°C with agitation of 50 rpm and 1.0 LPM flow of 0.22 μm filtered air; pH was maintained at 8.0 by automated addition of sterile 0.5 M K_2_CO_3_. Cell density (OD_600_) and nitrite concentration were measured daily by withdrawing a 5-ml sample from the reactor. Continuous culture for 7–10 days was alternated with batch culture (500 mL in 2-L Erlenmeyer flask) several times; each time an aliquot of the extracted genomic DNA was used to build a universal 16S rRNA cloning library for insert sequencing and analysis of sequence from 40 randomly picked clones (see text above) after each alternation. The alignments were inspected for single nucleotide polymorphisms. Once a single 16S rRNA sequence was observed across all clones in multiple subcultures, the culture was deemed pure and labeled “D1FHS.”

To inspect morphological homogeneity of the D1FHS culture, cells from 50 ml of a 10-day old culture were harvested by centrifugation (4,300 × *g*, 15 min). The cell pellet was washed twice with AMS medium and resuspended in 10 μl of AMS medium. A 2-μl aliquot of cell suspension was spread on a glass slide and covered with a cover slip to observe cell motility with a light microscope (Olympus, Japan) at 400× magnification. An 8-μl aliquot of cell suspension was heat-fixed on a slide for Gram staining (Sigma–Aldrich, USA) and observed using a light microscope (Olympus, Japan) at 1000× magnification.

For genome sequencing, ∼1.2 μg of gDNA were isolated from a 500-mL mid-exponential-phase culture. To obtain sufficient DNA for Pacific Biosciences (PacBio) single molecule sequencing, the purified genomic DNA was amplified by multiple displacement amplification (MDA) based on published protocols (Zhang et al., 2006). Briefly, 100 ng amounts of DNA were amplified in five MDA reactions (REPLI-g Mini, Qiagen). MDA products from each reaction (∼4 μg per reaction) were sequentially treated with 10 U Phi29 (Fermentas) and 100 U S1 nuclease (Thermo Fisher) followed by precipitation (SureClean, Bioline) and resuspension in sterile 18 Mω water. The identity of the amplified DNA was verified by PCR amplification with universal 16S rRNA primers. Half of the PCR product was digested with *Pst*I (Fermentas) to verify that they contained a single site as predicted and the remainder was directly sequenced with the amplification primers to verify that the sequence matched that from D1FHS. Following confirmation, the amplified DNA (∼15 μg total) was submitted for PacBio sequencing at the University of Delaware Genotyping and Sequencing Core Facility, University of Delaware. Sequence reads were assembled using RS_HGAP_Assembly protocol v.3 on a computational cluster maintained by the University of Delaware Center for Bioinformatics and Computational Biology. Completeness of the assembled genome was assessed by BLASTN and TBLASTN searches (CLC Main Workbench, Qiagen) using the D1FHS 16S rRNA sequence and a collection of 31 single-copy genes (Wu and Eisen, 2008). Biosample (SAMN04324281) and Bioproject (PRJNA305330) information has been submitted to the NCBI.

### HaoA Protein Sequence Analysis

HaoA protein sequences from *Nitrosococcus* species, including *N. oceani* strains C-107, C-27, AFC132, and AFC27, *N. watsonii* C-113, and *N. halophilus* Nc4, the deduced HaoA sequence from D1FHS and pertinent sequences of the “HAO” clade of octaheme cytochrome *c* proteins were aligned using MUSCLE and manually refined by comparison with previously published results from phylogenetic analyses including structural and protein sequence-analytical features (Klotz et al., 2008; Kern et al., 2011). N- and C-terminally extending sequences beyond the first and last heme-binding motif (CxxCH), respectively, were trimmed and the final alignment was subjected to a Bayesian inference of phylogeny using the BEAST package (v1.8.1 of BEAUti, BEAST and TreeAnnotator; FigTree v.1.4; Drummond et al., 2012). By utilizing unique sites, tree likelihoods (ignoring ambiguities) were determined for the alignment by creating a Monte–Carlo Markov Chain (10,000,000 generations) in three independent runs. The searches were conducted assuming an equal distribution of rates across sites, sampling every 1000th generation and using the WAG empirical amino acid substitution model (Whelan and Goldman, 2001). The resulting 10,000 trees (omitting the first 350 trees as burn-in) were used to construct a phylogenetic consensus tree.

### Average Nucleotide Identity between Bacterial Genomes

The Average Nucleotide Identity (ANI) calculated for pairwise compared genomes is an advanced method developed to identify species boundaries for bacteria and archaea (Konstantinidis and Tiedje, 2005). The species delineation cut-off point is an ANI of 94% (Konstantinidis and Tiedje, 2005; Goris et al., 2007), which corresponds to the traditional species cut-off of 70% using DNA–DNA hybridization (Wayne et al., 1987). The high quality draft genome sequence of strain D1FHS (single chromosome, no plasmid) was compared with the genome sequences of *N. halophilus* Nc4 (CP001798; plasmid: CP001799), *N. oceani* ATCC19707 (CP000127; plasmid: CP000126), *N. watsonii* C-113 [CP002086; plasmids: CP002087 & CP002088] as well as those of other Chromatiaceae, including *N. oceani* strain AFC-27 [ABSG00000000], *N. oceani* strain AFC132 [JPFN00000000], *N. oceani* strain C-27 [JPGN00000000], *Allochromatium vinosum* strain DSM 180 [CP001896; plasmids: CP001897 & CP001898], *Halorhodospira halophila* strain SL1 [CP000544], *Alkalimnicola ehrlichei* strain MLHE-1 [CP000453], *Thioalkalivibrio* sp. strains HL-EbGR7 [CP001339] and K90mix [CP001905; plasmid: CP00196] and *Halothiobacillus neopolitanus* strain c2 [CP001801] as previously done for the identification of *N. watsonii* as a species (Campbell et al., 2011). The ANI values reported in this paper were calculated using the tool developed and provided online by Luis M. Rodriguez-R and Kostas T. Konstantinidis^2^ using default parameters.

### Physiological Assays and Phenotypic Assessment

One-milliliter aliquots of mid-exponential-phase “D1FHS” pure culture were inoculated into 25 ml HEPES-buffered AMS-H media using 125 ml flasks and incubated in the dark at 30°C without shaking to determine growth as a function of time, salt and ammonium tolerance as well as optimum pH and temperature. A growth curve was estimated by measuring nitrite concentrations at eight times (days 0, 1, 3, 5, 6, 8, 10, and 12) after inoculation in AMS-H standard media [700 mM NaCl and 12.5 mM (NH_4_)_2_SO_4_ at a set pH of 8]. AMS-H media at eight different NaCl concentrations ranging from 100 to 1600 mM were used to determine the optimum NaCl concentration for the growth of D1FHS. Growth of the D1FHS culture was also tested at eight pH conditions (pH of 4, 5, 6,7, 7.5, 8, 9, and 10) and at five temperatures (4, 20, 28, 37, and 45C). Ammonium tolerance was examined by testing 10 ammonium concentrations [using (NH_4_)_2_ SO_4_] ranging from 0 to 600 mM. Cell cultures were monitored for nitrite production routinely. All tests were performed with three technical replicates and repeated with two or more biological replicates. Like *N. halophilus* Nc4 and unlike cultures of *N. oceani* C-107 and *N. watsonii* C-113 (Campbell et al., 2011), D1FHS did not grow on urea when provided as the sole source of ammonium (N, energy and reductant). Flagellar motility was observed under a light microscope at 1000× magnification.

## Results and Discussion

### Characterization of the Enrichment and Pure Isolate Cultures

PCR performed with metagenomic DNA extracted from D1 enrichment culture targeting at universal 16S rRNA, gamma 16S rRNA and gamma-*haoA* genes, yielded correctly sized fragments (universal 16S ∼1470 bp, gamma-16S ∼1500 bp, and gamma-haoA ∼1220 bp, respectively), indicating that the D1 enrichment culture contained gamma-AOB. Universal 16S rRNA gene sequences were analyzed based on BLASTN searches of the NCBI database; 29 of the 40 sequences were best hits to 16S rRNA gene sequences from *Nitrosococcus* species: 87–98% identity to *N. halophilus* Nc4; 93–98% identity to *N. oceani* C-107, and 93–98% identity to *N. watsonii* C-113. The rest of the sequences had best hits to sequences from *Mesorhizobium*, *Janibacter* sp., or uncultured bacteria.

Following the repeated enrichment procedure described in the experimental procedures that involved selection of colonies from semi-solid agar, alternating batch and continuous culture and the application of functional pressure by exposure to hydroxylamine and high sodium salt concentration, isolated genomic DNA from the final culture labeled “D1FHS” was, again, investigated using primers designed to detect universal 16S rRNA, gamma 16S rRNA and gamma-*haoA* genes and the PCR. Analysis of the obtained amplicon sequences using BLASTN consistently revealed highest identity with sequences from *N. halophilus* Nc4 and no clone had a best hit with sequences from bacteria outside of the *Nitrosococcus* genus.

### Genome Sequencing and Characteristics

PacBio-based high-throughput sequencing of D1FHS DNA produced 2,267,036,463 bp of sequence in 191,850 reads (N50 read length = 16,723 bp, mean read length = 11,816 bp). These were assembled into a high quality draft sequence containing one contig of 4,022,640 base pairs; the genome has a GC content of 50.7%. Homologs were detected for each of 31 universal single copy genes (Wu and Eisen, 2008) by TBLASTN searches and two copies of the D1FHS 16S rRNA sequence by BLASTN indicating that the assembled contig contains a complete bacterial genome. The sequence of the D1FHS genome was analyzed using the RAST annotation server (Aziz et al., 2008) and found to contain 4,128 protein-coding DNA sequences, 51 RNA genes including two 16S-23S-5S rRNA operons (RAST-ID: 6666666.126356). The genome sequence of D1FHS included genetic markers known from other *Nitrosococcus* genomes: the genome of D1FHS encodes a complete set of protein inventories required for ammonia-dependent chemolithotrophic growth, including ammonia monooxygenase (EC 1.14.99.39), hydroxylamine dehydrogenase (EC 1.7.2.6), cytochromes *c*554 and *c*_M_552 as well as nitrosocyanin, although the functions of cytochromes *c*554 and nitrosocyanin are still elusive (Stein et al., 2013). Based on the identification of signal peptides, all of these proteins operate and have access to their reactants within the periplasm or at the periplasmic side of the plasma membrane (Arp et al., 2007). Consistent with the inability of D1FHS to grow on urea, the genome did not encode urea hydrolase.

### Average Nucleotide Identity and HaoA Protein Phylogenetic Analysis

The ANI values for the comparison of the D1FHS genome with the genomes from Proteobacteria outside the genus *Nitrosococcus* were low as previously reported for the analysis of the *N. watsonii* C-113 genome (Campbell et al., 2011). ANI values calculated between the D1FHS genome and all available *Nitrosococcus* genomes including *N. halophilus* strain Nc4 (89.5%), *N. watsonii* C-113 (78.2%) and *N. oceani* (C-107, 78.4%; C-27, 78.2%; AFC27, 78.4%; AFC132, 78.2%) are significantly below the cut-off for the delineation of species (Konstantinidis et al., 2006), which suggests that strain D1FHS is representative of a species distinct from *N. halophilus*, *N. watsonii*, and *N. oceani* most closely related to *N. halophilus*. We propose to assign D1FHS as the type strain of *N. wardiae* spec. nov.

**FIGURE 2:**
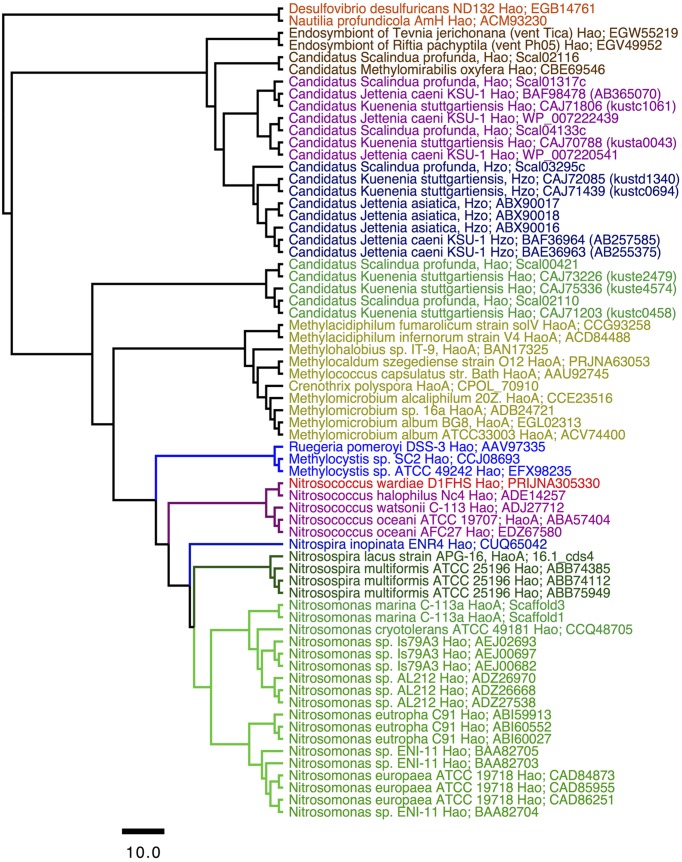
Consensus tree constructed after Bayesian inference of phylogeny from the MUSCLE alignments of sequences from octaheme cytochrome c proteins in the “Hao cluster” (Klotz et al., 2008; Kern et al., 2011) using the sequence from “reverse Hao” proteins as the out group (Hanson et al., 2013). The N- and C-terminal sequences outside the heme *c* binding motifs were eliminated from the final alignment that was subjected to Bayesian inference of phylogeny using the BEAST package (see *Experimental procedures*). Posterior probability values of all nodes were ≥0.99. Mean branch lengths are characterized by a scale bar indicating the evolutionary distance between the proteins (changes per amino acid position). The branches are annotated with labels indicating the source organism and the protein sequence accession number.

Phylogenetic analysis of protein sequences confirmed that the HaoA proteins from D1FHS and *N. halophilus* strain Nc4 are more closely related to one another than either is to HaoA proteins from strains of *N. watsonii* or *N. oceani* (**Figure 2**). Identical results were obtained from phylogenetic analysis of the other proteins implicated in ammonia-dependent chemolithotrophy (Arp et al., 2007; Stein et al., 2013): ammonia monooxygenase, cytochrome *c*_M_552 and nitrosocyanin (data not shown). The phylogram in **Figure 2** supports the conclusion that the ancestor of the *Nitrosococcus* genus diverged into two phylotypes that gave rise to *N. wardiae* spec. nov. and *N. halophilus* as well as *N. watsonii* and *N. oceani*. It was previously hypothesized that the *hao* genes encoded on the chromosome (soil methane-oxidizing) or a plasmid (marine sulfur-oxidizing) of Alphaproteobacteria and the *hao* genes in the genomes of betaproteobacterial AOB were obtained by horizontal transfer from Gammaproteobacteria (Klotz et al., 2008; Kern et al., 2011; Stein et al., 2011). The position of the Hao protein sequence from the “comammox” bacterium *Nitrospira inopinata* ERN4 capable of oxidizing ammonia all the way to nitrate (Daims et al., 2015) included in this analysis suggests that the *hao* gene cluster has been horizontally transferred from Gammaproteobacteria into the genomes of nitrite-oxidizing *Nitrospira* bacteria ancestral to the comammox lineage as well. Preliminary analysis of the D1FHS genome suggests that its genome is likely most representative of the ancestral *Nitrosococcus* genome; a detailed analysis of the genome is forthcoming.

### Growth-Physiological Characterization of D1FHS (**Table 4**)

**Table 4:** Classification and general features of *Nitrosococcus wardiae* D1FHS according to MIGS Recommendations (Chain et al., 2009).

MIGS ID	Property	Term	Evidence code
	Current classification	Domain *Bacteria*	IDA
		Phylum *Proteobacteria*	
		Class *Gammaproteobacteria*	
		Order *Chromatiales*	
		Family *Chromatiaceae*	
		Genus *Nitrosococcus*	
		Species *wardiae*	
		Strain D1FHS	
	Gram stain	Negative	IDA
	Cell shape	Coccus or short rod	IDA
	Motility	Motile	IDA
	Sporulation	Non-sporulating	IDA
	Temperature range	Mesophilic	IDA
	Optimum temperature	37°C	IDA
	Salinity	700 mM optimum	IDA
MIGS-22	Oxygen requirement	Aerobic	IDA
	Carbon source	Carbon dioxide (Calvin Benson Basham cycle)	IDA
	Energy source	Ammonia	IDA
	Energy metabolism	Chemolithotroph	IDA
MIGS-23	Isolation and growth conditions	Isolation after enrichment in ammonium minimal salts medium amended with hydroxylamine (AMS-H)	IDA
MIGS-6	Habitat	Marine sediment	IDA
MIGS-15	Biotic relationship	Free-living	NAS
MIGS-14	Pathogenicity	Non-pathogenic	NAS
	Biosafety level	1	NAS
MIGS-4	Geographic location	Yellow Sea/Jiaozhou Bay, China	IDA
MIGS-4.1	Latitude	36.067°N	IDA
MIGS-4.2	Longitude	120.230°E	IDA
MIGS-4.3	Depth	Sediment, 8 m depth	IDA
MIGS-4.4	Altitude	Not reported	NAS
MIGS-5	Sample collection time	October 26, 2008	IDA

*Evidence codes: IDA, inferred from direct assay (first time in publication); TAS, traceable author statement (i.e., a direct report exists in the literature); NAS: non-traceable author statement (i.e., not directly observed for living, isolated sample, but based on a generally accepted property for the species, or anecdotal evidence). These evidence codes are from a living isolate cultured by these authors*.

**FIGURE 3:**
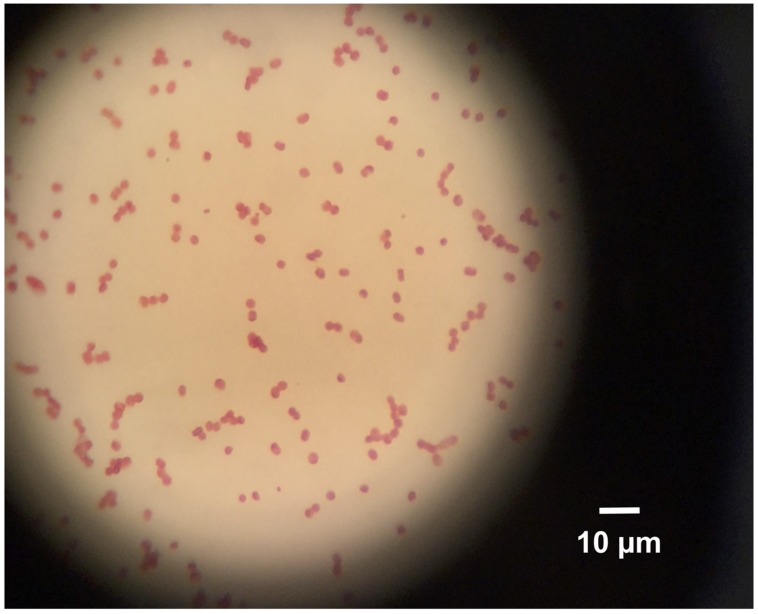
**Gram Stain, 1000× magnification using a light microscope**. Cell shape is short rods, arranged singly or in pairs.

In batch culture, cells of D1FHS routinely exhibited a lag time of 5 days before entering exponential growth phase. Concomitantly, cells started dividing and Gram-staining revealed that cells appear as short rods, arranged singly or in pairs but not in tetrads (**Figure 3**). The stain also confirmed the existence of only one cellular morphotype. In agreement with strains of other *Nitrosococcus* species, D1FHS contained intracellular membrane stacks, was lophotrichous and capable of flagellar motility (data not shown).

**FIGURE 4:**
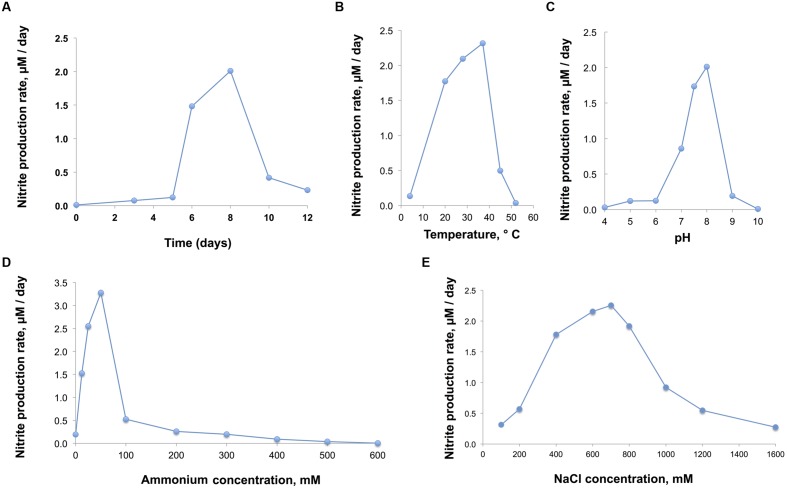
**(A)** Growth of D1FHS (assessed by maximum nitrite production rate) in AMS-H standard medium with 700 mM NaCl and 12.5 mM (NH_4_)_2_SO_4_ at pH 8, grown at 28°C in the dark. **(B,C)** Physiological tests: growth of D1FHS in AMS-H in the dark (assessed by maximum nitrite production rate) was characterized as a function of **(B)** temperature and **(C)** pH conditions. Data points are mean values calculated from three biological replicates. **(D)** Physiological tests: growth of D1FHS in AMS-H in the dark (assessed by maximum nitrite production rate) was characterized as a function of ammonium concentration. Data points are mean values calculated from three biological replicates. **(E)** Physiological tests: growth of D1FHS in AMS-H in the dark (assessed by maximum nitrite production rate) was characterized as a function of NaCl concentration. Data points are mean values calculated from three biological replicates.

The nitrite production rate was used to assess growth physiological characteristics of D1FHS (“optimum catabolism”), which was calculated based on a nitrite standard curve (calculated as μmol per L of media). The pH, temperature, NaCl and ammonium tolerances were determined by measuring the maximum nitrite production of log-phase cultures (day 5 to day 8; **Figure 4A**). Based on the observed maximum nitrite production rate, the optimum growth temperature for D1FHS is 37°C (**Figure 4B**), which is much higher than those reported (also based on nitrite production) for other strains of the genus *Nitrosococcus* (**Table 5**; Koops et al., 1990; Purkhold et al., 2000; Campbell et al., 2011). There is presently no published information for any *Nitrosococcus* strain about the need for maintenance energy at different temperatures. No growth (no changes in nitrite levels) was observed at 4 and 52°C, but D1FHS tolerated 45°C (**Figure 4B**). D1FHS was able to grow at a pH ranging from 5 to 9 with an optimum pH between 7.5 and 8.0 (**Figure 4C**), which is in the same range reported for other strains of the genus *Nitrosococcus* (**Table 5**). D1FHS was not able to tolerate ammonium concentrations higher than 300 mM, which represents a much lower tolerance than what is known for other *Nitrosococcus* strains (**Table 5**, **Figure 4D**; Koops et al., 1990; Purkhold et al., 2000; Campbell et al., 2011); consequently, the optimum ammonium concentration of 50 mM is also the lowest known optimum for all characterized strains of *Nitrosococcus*. D1FHS exhibited a NaCl tolerance similar to that of *N. halophilus* as it can grow at salt concentrations up to 1600 mM; the optimum salt concentration is 700 mM (**Figure 4E**). Taken together, *Nitrosococcus wardiae* D1FHS represents a new species that is characterized by a distinct growth physiology from other *Nitrosococcus* species.

**Table 5:** Characteristics of Type Strains of species in the genus *Nitrosococcus*.

	*Nitrosococcus wardiae* D1FHS	*Nitrosococcus halophilus* Nc4	*Nitrosococcus watsonii* C-113	*Nitrosococcus oceani* C-107
Habitat	Marine	Marine	Marine	Marine
16S rRNA gene clusters	2	2	2	2
Plasmids	0	1	2	1
Genome size	4,022,640 bp	4,079,427 bp	3,328,579 bp	3,481,691bp
G + C content	50.7%	51.6%	50.1%	50.4%
Cell shape and arrangement	Coccus or short rod	Coccus or short rod	Coccus or short rod	Coccus or short rod
	Singles and Pairs	Singles and Pairs	Singles and Pairs	Singles and Pairs
Temperature optimum	37°C	28–32°C	28–32°C	28–32°C
Salinity optimum	700 mM	800 mM	600 mM	500 mM
pH optimum	7.6–8.0	7.6–8.0	7.6–8.0	7.6–8.0
Ammonium tolerance	<300 mM	<600 mM	<1600 mM	<1200 mM
Ammonium optimum	50 mM	100 mM	100–200 mM	100 mM
Use of urea	–	–	+	+

### Emended Description of the Genus *Nitrosococcus*

*Nitrosococcus* (GenBank *Taxonomy* ID: 1227) synonym *Nitrosococcus* Winogradsky (Winogradsky, 1892); ex “*Micrococcus nitrosus”* Migula (Migula, 1900); ex *“Nitrosococcus nitrosus*” Buchanan (Buchanan, 1925; Editorial_Board, 1955; Commission, 1958). Name approved by Skerman et al. (1980), but the culture had been lost decades before. The etymology of *Nitrosococcus* is cataloged using digital optical identifiers as follows: *Genus*^3^, Family *Chromatiaceae*^4^ (Bavendamm, 1924), Order *Chromatiales*^5^ (Imhoff, 2005; List_Editor, 2005), Class *Gammaproteobacteria*^6^, phylum *Proteobacteria*^7^ (Garrity and Holt, 2001).

### Description of *Nitrosococcus wardiae* spec. nov.

#### Etymology

*Nitrosus* (Latin masculine adjective): nitrous; *coccus*: (Latin masculine adjective): sphere; *wardiae* (ward.i’ae. N.L. fem. gen. n. wardiae): of Ward, named after the American microbiologist Bess B. Ward for her pioneering work on the marine Nitrogen cycle including the study of AOB in the genus *Nitrosococcus*.

#### Locality as Well as Culture History

Collected on October 26, 2008, at station D1 in Jiaozhou Bay, a marginal sea bay in the Yellow Sea of China (36.067°N, 120.230°E) by Hongyue Dang and Martin G. Klotz. Maintained as enrichment culture D1 in the Klotz Lab (University of Louisville). Identified as a gammaproteobacterial AOB in an enrichment-culture in 2011 by Lin Wang and Martin G. Klotz and later purified using a hydroxylamine treatment regime by Lin Wang, Chee Kent Lim, and Martin G. Klotz (at UNC Charlotte).

Appearing as large cocci or very short rods. Cells contain a well-developed intra-cellular membrane system of an arrangement that appears as one stack of membrane vesicles packed mainly in the center of the cell. Polar flagella allow for motility, the genotype is chemotaxis-positive. Light sensitive; Strictly aerobic; Moderately alkaliphilic and mesophilic with an optimum growth temperature at 37°C; Grows in the presence of sodium salts between 200 to 800 mM with an optimum at 700 mM; Grows at ammonium concentrations below 300 mM with an optimum at 50 mM; The size of the genome is 4,022,640 base pairs with a GC content of 50.7%. No plasmid was identified. The genome of *N. wardiae* strain D1FHS^T^ (GenBank *Taxonomy ID:* 1814290) is presently being annotated and analyzed. MIGS information (Chain et al., 2009) to correlate the genotype with the environment of its isolation is provided in **Table 4**.

## Author Contributions

MGK conceived and designed the experiments; LW, CKL, HD, and TEH performed the experiments; LW, TEH, and MGK analyzed the data; and the manuscript was written by MGK with input from all authors.

## Conflict of Interest Statement

The authors declare that the research was conducted in the absence of any commercial or financial relationships that could be construed as a potential conflict of interest.

## Footnotes

1 http://www.ebi.ac.uk/Tools/msa/muscle/

2 http://enve-omics.ce.gatech.edu/ani/

3 doi://dx.doi.org/10.1601/nm.2107

4 doi://dx.doi.org/10.1601/tx.2070

5 doi://dx.doi.org/10.1601/nm.2069

6 doi://dx.doi.org/10.1601/tx.2068

7 doi://dx.doi.org/10.1601/tx.808
